# Rapid generation of gene-targeted EPS-derived mouse models through tetraploid complementation

**DOI:** 10.1007/s13238-018-0556-1

**Published:** 2018-06-13

**Authors:** Haibo Li, Chaoran Zhao, Jun Xu, Yaxing Xu, Chunmei Cheng, Yinan Liu, Ting Wang, Yaqin Du, Liangfu Xie, Jingru Zhao, Yanchuang Han, Xiaobao Wang, Yun Bai, Hongkui Deng

**Affiliations:** 1 Department of Cell Biology, School of Basic Medical Sciences, Peking University Stem Cell Research Center, State Key Laboratory of Natural and Biomimetic Drugs, Peking University Health Science Center and the MOE Key Laboratory of Cell Proliferation and Differentiation, College of Life Sciences, Peking-Tsinghua Center for Life Sciences, Peking University, Beijing, 100191 China; 2 Shenzhen Stem Cell Engineering Laboratory, Key Laboratory of Chemical Genomics, Peking University Shenzhen Graduate School, Shenzhen, 518055 China; 3 Peking University-Tsinghua University-National Institute of Biological Sciences Joint Graduate Program, College of Life Sciences, Peking University, Beijing, 100871 China; 4 BeiHao Stem Cell and Regenerative Medicine Translational Research Institute, Beijing, China

**Keywords:** tetraploid complementation, EPS, mouse model, CRISPR/Cas9

## Abstract

One major strategy to generate genetically modified mouse models is gene targeting in mouse embryonic stem (ES) cells, which is used to produce gene-targeted mice for wide applications in biomedicine. However, a major bottleneck in this approach is that the robustness of germline transmission of gene-targeted ES cells can be significantly reduced by their genetic and epigenetic instability after long-term culturing, which impairs the efficiency and robustness of mouse model generation. Recently, we have established a new type of pluripotent cells termed extended pluripotent stem (EPS) cells, which have superior developmental potency and robust germline competence compared to conventional mouse ES cells. In this study, we demonstrate that mouse EPS cells well maintain developmental potency and genetic stability after long-term passage. Based on gene targeting in mouse EPS cells, we established a new approach to directly and rapidly generate gene-targeted mouse models through tetraploid complementation, which could be accomplished in approximately 2 months. Importantly, using this approach, we successfully constructed mouse models in which the human interleukin 3 (*IL3*) or interleukin 6 (*IL6*) gene was knocked into its corresponding locus in the mouse genome. Our study demonstrates the feasibility of using mouse EPS cells to rapidly generate mouse models by gene targeting, which have great application potential in biomedical research.

**Electronic supplementary material:**

The online version of this article (10.1007/s13238-018-0556-1) contains supplementary material, which is available to authorized users.

## Introduction

Genetically modified mouse models are invaluable tools in biomedicine because they serve as powerful tools to study gene function, development and human diseases (Visigalli et al., 2010; Carido et al., 2014; Kenney et al., 2016). To generate mouse models with precise genetic modification, one major approach is gene targeting in mouse ES cells (Doyle et al., 2012), which are used to produce chimeric mice. By breeding these chimeric mice, postnatal mice with desired genetic modifications can be obtained through the germline transmission of gene-targeted ES cells. Notably, recent breakthroughs in genome editing, such as CRISPR/Cas9 technology, enable highly efficient genetic modifications in mouse ES cells (Cong et al., 2013), which further promote the applications of gene-targeted ES cells in generating mouse models.

Although gene targeting of mouse ES cells has been widely used for generating mouse models in recent decades (Tang et al., 2009; Rappaport and Johnson, 2014), one major limitation of this approach is that it is difficult to maintain the germline competence of gene-targeted ES cells using conventional ES culturing medium. For instance, long-term culture of ES cells in 2i medium (Ying et al., 2008), a well-established condition for maintaining ES self-renewal, cannot maintain the genetic and epigenetic stabilities of ES cells in the long term, resulting in karyotype abnormalities and impaired methylation of imprinted genes (Choi et al., 2017; Yagi et al., 2017). These changes eventually lead to reduced developmental potency of ES cells, which impairs their chimeric ability and germline competence. Another problem of current approaches to generate mouse models using gene-targeted ES cells is that it is time-consuming and labor-intensive: it usually takes 9 months to 1 year to obtain the mouse model by breeding. To solve these problems, there is a high demand for developing a robust and fast approach to generate mouse models with sophisticated and precise genetic modifications.

Recently, we have developed a new culture condition that permits the generation of extended pluripotent stem (EPS) cells with embryonic and extraembryonic developmental potency (Yang et al., 2017). One major feature of mouse EPS cells is their robust chimeric ability, as single mouse EPS cells show widespread chimeric contribution to both intraembryonic and extraembryonic lineages. More importantly, these cells have the power of tetraploid complementation, and EPS-derived postnatal mice can be generated by injecting single mouse EPS cells through tetraploid complementation. The superior developmental potency and chimeric ability of EPS cells raise the possibility of using EPS cells to develop a robust and rapid method to generate genetically modified mice through tetraploid complementation, which remains to be explored.

Here, we established a new approach to directly generate gene-targeted mouse models with the integration of EPS cells and tetraploid complementation. We demonstrated its feasibility by generating genetically modified mouse models in which the human *IL3* or *IL6* gene was knocked into its corresponding locus in the mouse genome. This novel approach addresses a major barrier to construct mouse models with comprehensive genetic modifications, greatly decreasing the time to generate genetically modified animals.

## Results

### Maintenance of genetic and epigenetic stability of EPS cells after long-term culturing

**Figure 1:**
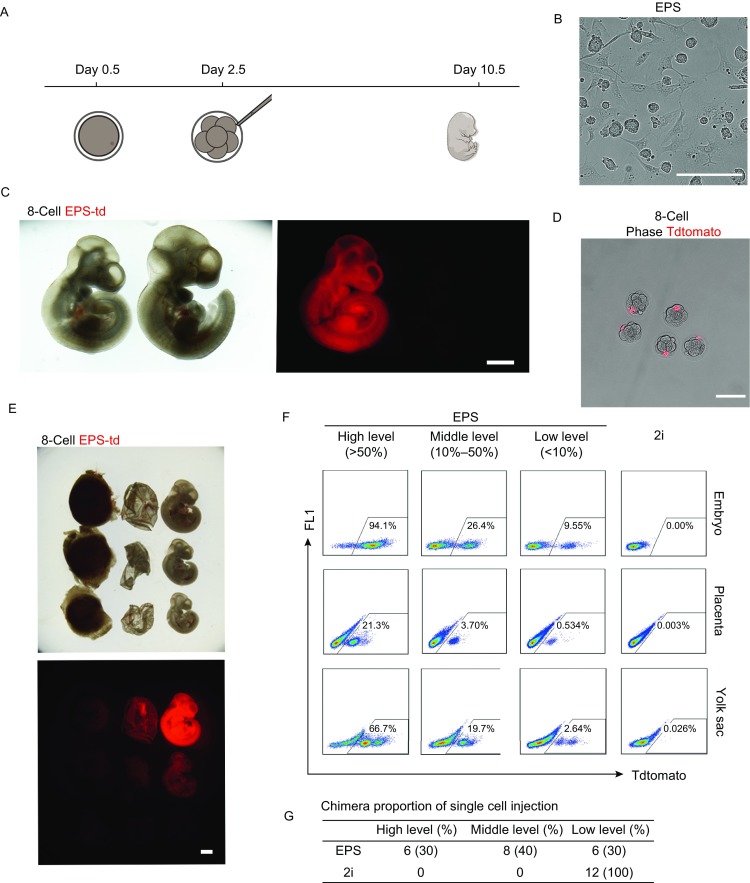
**EPS cells have superior efficiency in generating chimeras**. (A) Strategy of injecting mouse EPS cell into 8-cell embryos for analysis. Eight-cell embryos were injected with 8–15 EPS cells, and conceptuses were examined at E10.5. (B) The colonial morphology of EPS cells. Scale bars, 50 μm. (C) Injection of multiple EPS cells generated high-level chimeras. Left, E10.5 chimeric conceptus. Right, negative control. Eight to fifteen EPS-Td cells were injected into 8-cell embryos, and the Td signal was analyzed in E10.5 conceptuses. Td, Tdtomato fluorescent signal. Scale bars, 1 mm. (D) Diagrams showing the injection of single EPS-Td cells into 8-cell embryos. Scale bars, 50 μm. (E) Representative images showing the chimerism of single EPS-td derivatives in the embryo, placenta and yolk sac from an E10.5 conceptus. From top to bottom: high, middle and low levels of chimerism. Scale bars, 1 mm. (F) Representative FACS analysis of the percentages of single EPS derivatives in an E10.5 conceptus. Single 2i-ES cells were used as the control. (G) Table summary of FACS analysis of chimerism in E10.5 conceptus

To confirm the chimeric ability of EPS cells, we injected multiple or single EPS cells into 8-cell embryos and transferred these embryos *in vivo* (Fig. 1A and 1B). On day 10.5 of pregnancy, the surrogate mothers were sacrificed to determine the ratio of chimerism in the embryos. As Figure 1C shows, EPS cells produced a significantly high proportion of chimeras. In particular, a single EPS cell (Fig. 1D) produced almost the entire mouse (Fig. 1E–G). As a control, ES cells cultured under the 2i condition (2i-ES) did not produce any detectable single-cell chimerism (Fig. 1F and 1G). These results were consistent with our previous observations that mouse EPS cells have superior chimeric ability compared to conventional 2i-ES cells.

**Figure 2:**
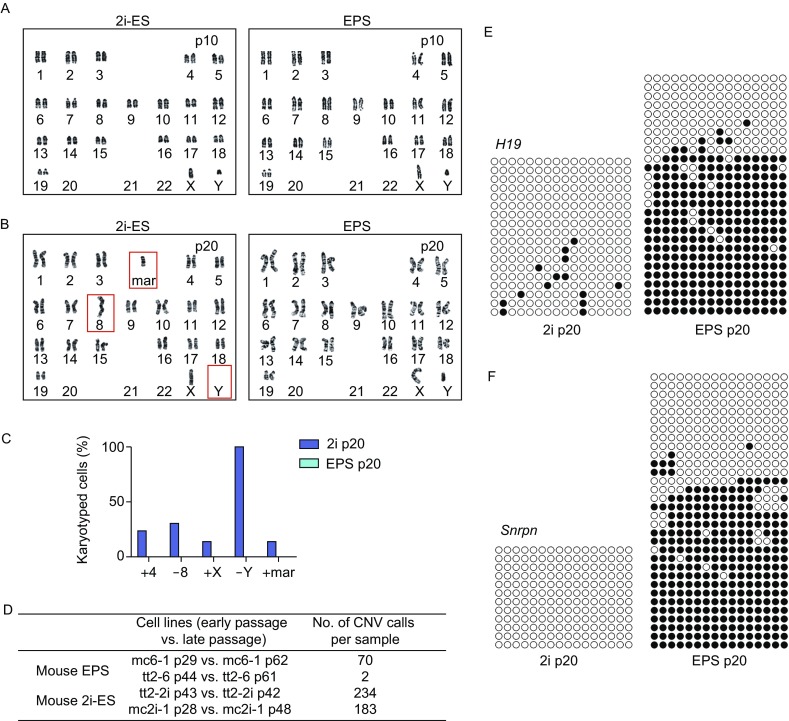
**EPS cells are more stable than 2i cells at both the genetic and epigenetic levels**. (A and B) Karyotype analysis of 2i-ES cells and EPS cells. Cells were collected at the indicated passage. (C) Percentage of cells with abnormal karyotype in 2i-ES cells and EPS cells. 30 2i-ES cells and 30 EPS cells at metaphase were analyzed. (D) CNVs in EPS cells and 2i-ES cells analyzed by CGH profiling. (E and F) DNA methylation status of *H19* (E) and *Snrpn* (F) in 2i-ES cells and EPS cells at passage 20. DNA methylation profiles were assayed by the bisulfite sequencing assay. Each line represents an individual clone allele. Each circle within the row represents a single CpG site (open and closed circles represent unmethylated and methylated CpGs, respectively)

To explore the potential factors responsible for the difference in chimeric ability between EPS and 2i-ES cells, we first focused on analyzing the genome stability, which was reported to affect the developmental potency of pluripotent cells (Plasschaert and Bartolomei, 2014). To this end, we examined the karyotypes of both EPS and 2i-ES cells at different passages. Both 2i-ES and EPS cells had normal karyotypes at passage 10 (Fig. 2A). However, after further passaging, the karyotype of 2i-ES cells showed significant abnormalities. 2i-ES cells completely lost the Y chromosome, and some cells lost chromosome 8 (Fig. 2B). In addition, several 2i-ES cells had extra chromosomes, such as chromosome 4, chromosome X and the mar chromosome (Fig. 2C). In contrast, the karyotype of EPS cells remained normal (Fig. 2B and 2C). To further analyze the genetic stability, we examined the copy number variation (CNV) in these two cell types at different passages, which indicates the rearrangement of the genome. Compared to the original cells at early passage, EPS cells showed relatively low CNV mutation. Surprisingly, a high CNV mutation rate was observed in 2i-ES cells (Fig. 2D). Collectively, these results indicate that mouse EPS cells possess genetic stability compared to mouse 2i-ES cells after long-term culturing.

We next attempted to investigate the DNA methylation of imprinted genes in EPS and 2i-ES cells, which would reflect the stability on the epigenetic level. We selected *H19* and small nuclear ribonucleoprotein N (*Snrpn*) for analysis because dysregulation of these two loci is reported to affect the developmental potency of pluripotent cells (Plasschaert and Bartolomei, 2014). After long-term culturing, EPS cells showed stable maintenance of DNA methylation at *H19* and *Snrpn*. In contrast, 2i-ES cells almost completely lost DNA methylation at these two loci (Fig. 2E and 2F). These results further suggest that mouse EPS cells are epigenetically stable compared to 2i-ES cells. The stability of EPS cells at both the genetic and epigenetic levels may contribute to the robust developmental potency of these cells.

### Replacement of *Il3* or *Il6* gene with its human counterpart in EPS cells using the CRISPR/Cas9 technique

**Figure 3:**
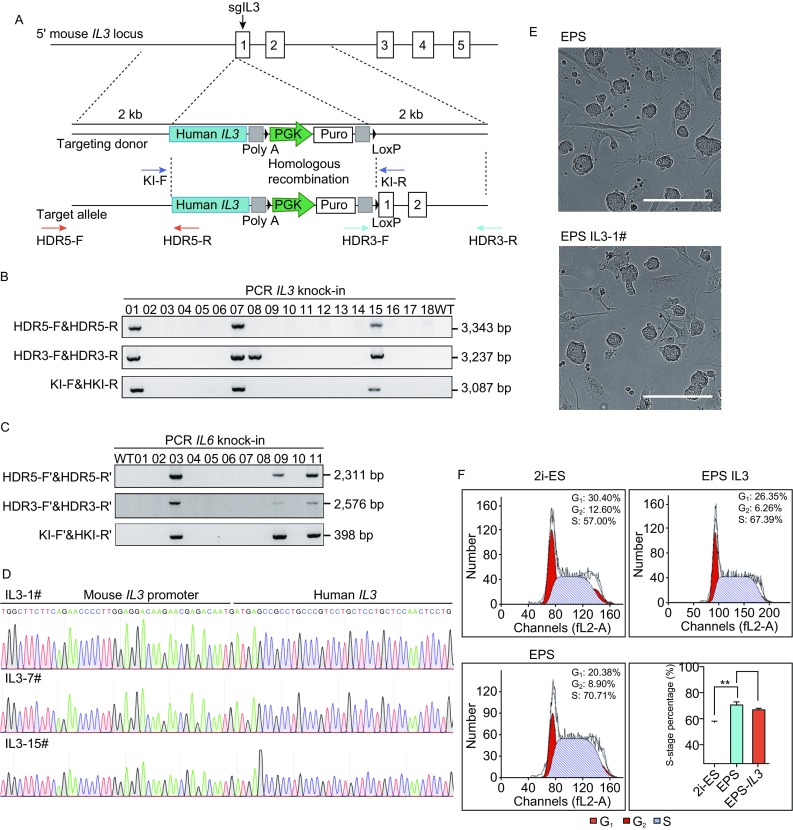
**Generation of human** ***IL3*** **or** ***IL6*** **gene knock-in EPS cells**. (A) Diagrams of generating the human *IL3* gene knock-in EPS cells. Primers for knock-in detection were indicated as pairs of arrows. (B and C) Representative images showing identification of successful knock-in of human *IL3* (B) or *IL6* (C) genes into its corresponding locus in mouse EPS cells. EPS cells without gene targeting were used as the wild-type control. WT, wild type. (D) Sequencing results of the promoter sites at the mouse *Il3* locus showing the correct insertion of the human *IL3* gene. (E) Representative images showing the morphology of EPS and EPS-*IL3* clones. Scale bars, 50 μm. (F) Representative FACS analysis of the cell cycle of 2i-ES cells, EPS cells and EPS-*IL3* cells. The percentages of cells at G_1_, S and G_2_ are shown on the right side of each chart. Bar chart shows the S-stage percentage of each cell type. Error bars indicate SEM (*n* = 3). Significant differences between values of 2i-ES cells and EPS cells were found by *t*-test (**P < 0.01)

The superior chimeric ability and genetic/epigenetic stability of mouse EPS cells make them promising tools for generating mouse models. To test this possibility, we first attempted to replace mouse genes with human genes in mouse EPS cells by the CRISPR/Cas9 technique. IL3 and IL6 are essential for the development of hematopoietic stem cells into macrophages and B cells (Rongvaux et al., 2013), and expression of the human IL3 or IL6 product in mice promotes the reconstitution of part of the human immune system when transplanting human hematopoietic stem cells (HSCs) into immune-deficient mice (Willinger et al., 2011; Yu et al., 2017). Importantly, these loci are difficult to precisely target in mouse cells because the presence of multiple off-target sites. Therefore, the use of mouse EPS cells in generating human *IL3* and human *IL6* knock-in cell lines provides an opportunity to examine the application potential of mouse EPS cells in generating mouse models, especially those that are difficult to establish using conventional approaches. To target the mouse *Il3* and *Il6* loci by CRISPR/Cas9 technology, we first designed 3–4 guide RNAs (gRNAs) for each locus and selected the one with the highest efficiency of targeting the *Il3* or *Il6* locus. The human *IL3* and human *IL6* genes were designed to be inserted into the mouse *Il3* and mouse *Il6* locus following the corresponding mouse *Il3* and mouse *Il6* promoters, respectively (Figs. 3A and S2). As a result, the human *IL3* and human *IL6* gene expression was driven by the endogenous mouse *Il3* and mouse *Il6* regulatory elements, which could facilitate the development of human immune cells in humanized mouse models. After delivering the donor fragment and gRNA/Cas9 vector into mouse EPS cells, a large number of clones were obtained by puromycin selection, which were further picked for expansion and genomic analysis. The targeting efficiencies were 16.7% for *IL3* (Fig. 3B) and 27.3% for *IL6* (Fig. 3C), as revealed by genomic PCR. These clones were also checked by DNA sequencing (Fig. 3D). And we did not detect any off-target effects in the predicted off-target sites in these cell lines (Fig. S3). Importantly, we did not find any significant changes in the characteristics of the cells after gene targeting (Figs. 3E, 3F and S4). Collectively, these results suggest the robustness of performing gene targeting in mouse EPS cells.

### Generation of high-level chimeric mice using human *IL3* or *IL6* knock-in EPS cell lines

**Figure 4:**
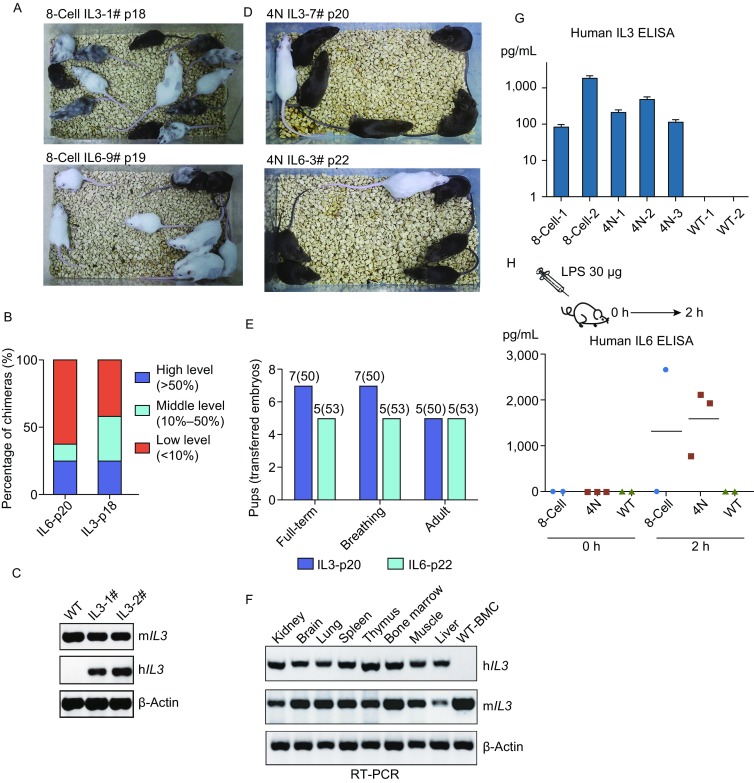
**Analysis of** ***IL3*** **and** ***IL6*** **8-cell- and tetraploid-derived mice**. (A) Chimeras generated by injecting *IL3* or *IL6* EPS cells into 8-cell embryos. Cells were injected into 8-cell embryos at the indicated passage. (B) Bar chart shows the percentage of chimeras generated by 8-cell embryo injection. (C) RT-PCR analysis of human *IL3* (h*IL3*) and mouse *Il3* (m*IL3*) expression in bone marrow cells isolated from chimeras generated by 8-cell embryo injection. The negative-control cells were collected from wild-type mice. (D) Representative images showing *IL3*- or *IL6*-targeted EPS cell-derived mice through tetraploid complementation. Cells were injected into tetraploid blastocysts at the indicated passage. (E) Bar chart shows the proportion of full-term, breathing, adult mice derived from transferred embryos in the tetraploid complementation assay. (F) RT-PCR analysis of h*IL3* and m*IL3* expression in different tissues of *IL3*-targeted EPS cell-derived tetraploid mice. (G) ELISA measurement of hIL3 expression in peripheral blood isolated from chimeras generated by 8-cell embryo injection of *IL3*-targeted EPS cells (8-cell-1 and 8-cell-2), as well as *IL3*-targeted EPS cell-derived mice by tetraploid complementation (4n−1, 4n−2 and 4n−3). The negative control was wild-type ICR mice. Error bars indicate SEM (*n* = 3). Data were analyzed by *t*-test. (H) ELISA measurement of human IL6 expression in peripheral blood from chimeras generated by 8-cell embryo injection of *IL6*-targeted EPS cells (8-cell), as well as *IL6*-targeted EPS cell-derived mice by tetraploid complementation (4N). LPS was used to stimulate human IL6 secretion. Each mouse was treated by 30 μg LPS, and peripheral blood was collected after 2 h. Each dot represents 1 mouse. Horizontal bars indicate mean values

After obtaining the human *IL3* and human *IL6* knock-in EPS cell lines, we tried to generate chimeric mice by injecting these cell lines into 8-cell embryos. Notably, the genetically modified *IL3* and *IL6* knock-in EPS cells still retained their superior chimeric ability, and high-level chimeric mice were obtained (Fig. 4A and 4B). Among these chimeric mice, 3/12 *IL3* knock-in and 2/8 *IL6* knock-in mice were generated almost exclusively from donor cells, as judged by coat color. To confirm the presence of mouse cells expressing human *IL3* product, we isolated bone marrow cells from chimeric mice with high chimerism at 8-week age. Using reverse transcription PCR (RT-PCR), we found that the human *IL3* mRNA transcript was well expressed in the corresponding position (Fig. 4C). Therefore, these data indicate that the human *IL3* and human *IL6* knock-in EPS cell lines are efficient in generating chimeric mice.

### Direct generation of human *IL3* or *IL6* knock-in mice through tetraploid complementation

The success of generating human *IL3* or *IL6* knock-in chimeric mice led to the question of whether human *IL3* and human *IL6* knock-in EPS cell lines would also permit the direct generation of human *IL3* and human *IL6* knock-in mice by tetraploid complementation. Therefore, we tried to inject these cells into tetraploid mouse blastocysts. In three independent experiments, we obtained 7 human *IL3* knock-in EPS cell-derived mice and 5 human *IL6* knock-in EPS cell-derived mice (Fig. 4D and 4E) after injecting 50 and 53 blastocysts, respectively. However, the control group of human *IL3* knock-in ES cells was injected into 46 tetraploid blastocysts. At 10.5 days, we observed only the placenta and no embryos were observed (Fig. S5). To examine the expression of human *IL3* product, several human *IL3* knock-in mice were sacrificed for analysis at 8 weeks of age. As Figure 4F shows, we detected robust human *IL3* gene expression in the bone marrow and observed a similar pattern of expression for both mouse and human *IL3* mRNA in all analyzed organs by RT-PCR. In addition, ELISA confirmed that peripheral blood indeed contained human IL3 protein (Fig. 4G). To examine the protein level of human IL6 product in the human *IL6* knock-in mice, we performed lipopolysaccharide (LPS) activation experiments to see whether human IL6 production could be stimulated by LPS treatment. Two hours after injection of 30 µg LPS, peripheral blood was drawn for ELISA. The results showed that human IL6 secretion reached the level of 2,000 pg/mL, which was much higher than that without LPS activation (2 pg/mL) (Fig. 4H). Collectively, these results demonstrated that humanized *IL3* knock-in mice or *IL6* knock-in mice can be generated through tetraploid complementation of EPS cells and that these mice have the correct pattern of IL3 and IL6 expression.

## Discussion

In this study, we established an EPS cell-based approach for efficiently generating mouse models with precise genetic modifications. This approach combines EPS cells, tetraploid complementation and CRISPR/Cas9 technology, which permits the production of mouse models that are precisely genetically modified within 2 months. Using this new approach, we successfully generated mouse models that replaced the mouse *Il3* and *Il6* genes with their human counterparts. These results demonstrate the feasibility of using our approach to efficiently and rapidly create mouse models, especially those that are difficult to produce using conventional gene-targeting strategies.

One unique advantage of using EPS cells for generating genetically modified mouse models is their stability at high passages. It is well known that the chromosomal make-up of ES cells predicts their developmental potency (Choi et al., 2017; Li et al., 2017; Yagi et al., 2017). Notably, mouse EPS cells still showed a normal karyotype after 20 passages *in vitro*, whereas 2i-ES cells exhibited significant abnormalities (Fig. 2B). More importantly, our comparative genomic hybridization (CGH) analysis further showed that mouse EPS cells showed significantly lower CNV mutations after long-term culturing (20–30 passages) compared to 2i-ES cells (Fig. 2D). These results strongly indicate the improved genetic stability of mouse EPS cells compared with 2i-ES cells. In addition to genetic stability, epigenetics also greatly affect the developmental potency of pluripotent cells. Epigenetics can be altered by long-term culture or *in vitro* manipulation (Choi et al., 2017; Yagi et al., 2017). Importantly, mouse EPS cells still retained normal imprinting marks in the *H19* and *Snrpn* loci after long-term culturing, suggesting their epigenetic stability (Fig. 2E and 2F). In contrast, long-term-cultured 2i ES cells lost DNA methylation in these loci (Fig. 2E and 2F). Accordingly, the genetic and epigenetic stability of mouse EPS cells contributes to the maintenance of their developmental potency after gene targeting, which makes it feasible to use these genetically modified cells for efficient tetraploid complementation.

Remarkably, the combination of gene targeting in EPS cells and tetraploid complementation established a rapid way to generate animal models with precise and sophisticated genetic manipulations. Tetraploid complementation is a functional assay to rigorously evaluate the developmental potency of pluripotent stem cells (Zhao et al., 2009). Because complete pluripotent cell-derived mice can be directly obtained through tetraploid complementation, this method bypasses the requirement of mouse breeding, which is needed for generating mouse models by conventional gene-targeted ES cells or by direct genetic modification of zygotes (Yang et al., 2014). In principle, our new approach could significantly decrease the time required for generating mouse models to 2 months. This unique advantage would be particularly useful for generating sophisticated mouse models with genetic modifications in the future.

In summary, our novel approach enables rapid and efficient generation of mouse models. The use of tetraploid complementation can greatly shorten the time required for generating genetically modified mouse models. In future studies, it will be important to test whether this platform could be applied to genetic modification of special strains, such as NSG (NOD Scid Gamma) mice (Ito et al., 2002), which would be beneficial for producing humanized mice with highly efficient human cell engraftments and robust reconstitution of the human immune system. In short, our approach could provide an opportunity to advance the generation and application of mouse models in the future.

## Materials and methods

### Mice

The C57BL/6 and ICR mice were raised in a specific pathogen free (SPF) animal facility by the Institutional Animal Care and Use Committee of Peking University Health Center. Experiments with ICR and C57 mice were performed in males at 8 weeks of age and females at 4 weeks of age.

### Animal treatment

Females treated with 10 IU pregnant mare serum gonadotropin (PMSG) and 48 h later with 10 IU human chorionic gonadotropin (hCG) were mated with male mice. Only female mice with vaginal plugs were determined to have successfully copulated, and this was regarded as day 0.5 of pregnancy. Eight-cell embryos and blastocysts were collected on days 2.5 and 3.5 of pregnancy. Female mice were mated with male mice after vasoligation. Only female mice with vaginal plugs the day after mating were determined to be 0.5 d pseudo-pregnant mice to be embryo transfer receptors.

### Culture of mouse embryos

For embryo culture, mouse embryos were kept in 20 µL drops of EmbryoMax KSOM embryo culture (Millipore, MR-020P-5F) covered with mineral oil (Sigma-Aldrich, M8691) in a humidified incubator under 5% CO_2_ at 37 °C. During the process of microinjection, embryos were placed in 10 µL EmbryoMax M2 medium (Millipore, MR-015-D) covered with mineral oil.

### Culture of mouse EPS cells

mEPS cells were derived directly from blastocysts of F1 hybrids between C57 and 129 mice. Blastocysts were cultured on feeder cells for 4–5 days in EPS medium, which contained 120 mL DMEM/F12 (Thermo Fisher Scientific, 11330-032), 120 mL neurobasal (Thermo Fisher Scientific, 21103-049), 1.25 mL N2 supplement (Thermo Fisher Scientific, 17502-048), 2.5 mL B27 supplement (Thermo Fisher Scientific, 12587-010), 1% GlutaMAX (Thermo Fisher Scientific, 35050-061), 1% nonessential amino acids (Thermo Fisher Scientific, 11140-050), 0.1 mmol/L β-mercaptoethanol (Thermo Fisher Scientific, 21985-023), penicillin-streptomycin (Thermo Fisher Scientific, 15140-122), and small molecules and cytokines added to the N2B27 medium at the following final concentrations: 10 ng/mL recombinant human LIF (10 ng/mL; Peprotech, 300-05), CHIR 99021 (3 µmol/L; Tocris, 4423), (S)-(+)-dimethindene maleate (2 µmol/L; Tocris, 1425) and minocycline hydrochloride (2 µmol/L; Santa Cruz Biotechnology, sc-203339). Outgrowths were trypsinized and passaged every 2–3 days for further analysis. EPS-td cells were obtained by EF1α-Tdtomato lentivirus infection.

### Cell cycle analysis by DNA flow cytometry

Cells were collected in tubes and washed three times to discard the remains of cell culture medium. Pre-cooled 70% alcohol was added dropwise to the cells, and the tube was left at 4 °C for more than 18 h. The tube was then centrifuged, and the cells were re-suspended with 20 µg/mL PI and 50 µg/mL RNase. After 15 min staining, the samples were analyzed on a BD FACSCalibur machine.

### Construction of CRISPR/Cas9 vector and donor template

Targeting-guide RNAs were designed based on the software available from the website http://crispr.dbcls.jp/. We chose 3–4 gRNAs for each gene targeting. Overhangs were added to allow ligation to the pX330-U6-Chimeric_BB-CBh-hSpCas9 vector. pX330-U6-Chimeric_BB-CBh-hSpCas9 was a gift from Feng Zhang (Addgene plasmid #42230) (Cong et al., 2013). Two oligonucleotides for each target were synthesized by Rubiotech and annealed with the gradient descent method. The pX330 vector was digested by BbsI (NEB) for 16 h at 37 °C. The *IL3* or *IL6* gRNA-pX330 vector was constructed by ligating the gRNA annealed product and the pX330 digested product. The human *IL3* and *IL6* gene clones were bought from Origene and had overhangs added to them by PCR for the next infusion step. The homology arms covering the 2 kb upstream and downstream of the target gene were obtained by PCR from the mouse genome. All fragments were constructed by an In-Fusion kit (Takara) to make an *IL3*-LoxP-PGK-Puro-LoxP or *IL6*-LoxP-PGK-Puro-LoxP donor vector. The donor vector was linearized by NotI overnight at 37 °C. We used ethanol precipitation to purify the *IL3* or *IL6* gRNA-pX330 vector and donor template before electrical transfection.

### EPS cell culture and electrical transfection

EPS cells were cultured on 2 × 10^6^ feeder cells in a Falcon Multiwell 6-well plate supplemented with EPS medium. For electrical transfection, a total of 20 µg of the appropriate DNA fragment was transfected into 2 × 10^6^ EPS cells using a LONZA P3 Primary Kit. The 20 µg of DNA fragment contained 10 µg *IL3* or *IL6* gRNA-pX330 vector and 10 µg linearized donor template. Forty-eight hours after transfection, selection was performed in 500 ng/mL puromycin (Gibco, A11138-02). Clones were picked up after another 48 h.

### Establishment of *IL3* or *IL6* EPS cell lines

Clones were picked and passaged by 0.05% trypsin-EDTA and were examined by PCR. Genomic PCR was performed using PrimeSTAR® HS DNA Polymerase with GC Buffer (Takara, R044B). Primers of h*IL6*-insert: HDR5-insert forward, TGGATGTATGCTCCCGACTT, HDR5-insert reverse, TTCTGCCAGTGCCTCTTTGC, a total of 2,311 bp; HDR3-insert forward, CTCTTTACTGAAGGCTCTTTACTATTGCT, HDR3-insert reverse, TCCACTTCTGACCCTCACTCCTT, a total of 2,576 bp; h*IL6*-insert forward, CACAGACAGCCACTCACCTC, h*IL6*-insert reverse, AGGCTGGCATTTGTGGTTGG, a total of 398 bp. Primers of h*IL3*-insert: HDR5-insert forward, CATTAGCACCAGAACCTCCCTCAG, HDR5-insert reverse, TCACCGTCCTTGATATGGATTGG, a total of 3,343 bp; HDR3-insert forward, CTACGAGCGGCTCGGCTTCA, HDR3-insert reverse, CCTGTCATGGGTCATCTTGGACAAT, a total of 3,502 bp; h*IL3*-insert forward, TAACCATGTGCCAGAATGCCTACC, h*IL3*-insert reverse, TGGAACCCAAGAATATCCCAAAGC, a total of 3,087 bp. After gel electrophoresis, the correct clones were sequenced.

### Chimeric assay of multiple-cell microinjection

For EPS cell injection, EPS cells were trypsinized by 0.05% trypsin-EDTA and filtered through a cell strainer (40 µm). Eight to ten EPS cells were microinjected into 8-cell stage embryos or blastocysts. Then, 15–20 8-cell embryos were transferred to the oviduct of E0.5 pseudo-pregnant females, and 10–15 blastocysts were transferred to the uterine horn of E2.5 pseudo-pregnant females.

### Assay of tetraploid complementation

Two-cell embryos were collected from ICR 1.5 d pregnant mice cultured in KSOM medium in a humidified incubator under 5% CO_2_ at 37 °C. The cell fusion program was carried out by an electrofusion device (BLS, CF-150/Bsp) to produce tetraploid embryos by electrofusion. Tetraploid embryos were washed by M2 (Sigma) and KSOM. Ten to fifteen EPS cells were injected into tetraploid blastocysts, and 10–15 embryos were transferred to the uterus of ICR 2.5 d pseudo-pregnant recipients.

### ELISA

For the *IL6* ELISA, LPS (30 µg per mouse, InvivoGen) was injected into *IL6* and wild-type mice. The plasma was collected from the orbit after 2 h. For *IL3* ELISA, plasma was collected directly from *IL3* and wild-type mice. Cytokines in mouse plasma were measured using a Human IL-6 ELISA Kit (Dakewe, DKW12-1060-096) and a Human IL-3 ELISA Kit (Sigma, RAB0294) following the manufacturer’s instructions.

### DNA methylation bisulfite treatment assay

Genomic DNA was extracted from cells at the indicated passages according to the instructions of the Blood and Tissue Kit (Qiagen). The genomic DNA was treated with bisulfite according to the manufacturer’s instructions (MethylCode™ Bisulfite Conversion Kit, Life, MECOV50). We used nested PCR with bisulfite-treated DNA in the first round. The first-round PCR used the outside primers, whereas the second-round PCR used the inside primers. The first round of PCR of *H19* consisted of 94 °C for 6 min, 35 cycles of 94 °C for 1 min, 55 °C for 2 min, and 72 °C for 3 min, and a final extension at 72 °C for 5 min. For the second round of PCR of H19, 1 µL of the first-round sample was used: denaturation at 94 °C for 5 min, 30 cycles at 94 °C for 40 s, 55 °C for 45 s, and 72 °C for 50 s, and a final extension at 72 °C for 5 min. The primers used for bisulfite sequencing were *H19* outside forward: GAGTATTTAGGAGGTATAAGAATT; outside reverse: ATCAAAAACTAACATAAACCCCT; inside forward: GTAAGGAGATTATGTTTATTTTTGG; inside reverse: CCTCATTAATCCCATAACTAT; The first round of PCR of *Snrpn* consisted of denaturation at 94 °C for 6 min and 35 cycles at 94 °C for 30 s, 1 min at 55 °C, and 1 min at 72 °C. For the second round of PCR of *Snrpn*, 1 µL of the first-round sample was used, and the conditions for the PCR were the same. The primers used for bisulfite sequencing were *Snrpn* outside forward: TATGTAATATGATATAGTTTAGAAATTAG, outside reverse: AATAAACCCAAATCTAAAATATTTTAATC, inside forward: AATTTGTGTGATGTTTGTAATTATTTGG, inside reverse: ATAAAATACACTTTCAQCTACTAAAATCC. The PCR product was ligated with a pEASY-Blunt vector (pEASY-Blunt Simple Cloning Kit, TransGen Biotech, CB111-01) and was sequenced by Rubiotech.

### Immunofluorescence analysis

Cells were fixed with 4% paraformaldehyde for 15 min and washed by PBS three times. Then, they were permeabilized with PBS containing 0.1% Triton X-100 and 3% donkey serum for 1 h. Cells were incubated in primary antibody overnight at 4 °C and secondary antibody at room temperature for 1 h. Cells were washed by PBS with 0.1% Tween-20 three times after every step. The nuclei were stained with DAPI (Roche Life Science, 10236276001). The antibodies were anti-FOXA2 (1:200; ab60721; Abcam), anti-b-III TUBULIN (1:300; Santa Cruz, sc-80016), anti-NANOG (1:100; Abcam, ab80892), anti-CDX2 (CDX2-88; Biogenex, AM392), anti-GATA3 (1:200; Santa Cruz, sc-268), anti-EOMES (1:200; Abcam, AB23345), and anti-OCT4 (1:200; Santa Cruz, sc-5279), anti-SOX2 (1:200; Santa Cruz, sc-17320).

## Electronic supplementary material

Below is the link to the electronic supplementary material.

Supplementary material 1 (PDF 296 kb)
