# Supplementary material for: Rapid generation of gene-targeted EPS-derived mouse models through tetraploid complementation
Source: Protein Cell. 2018 Jun 13;10(1):20–30. doi: 10.1007/s13238-018-0556-1 (PMC6321812; doi:10.1007/s13238-018-0556-1)
Supplement: Supplementary file 1 — Supplementary material 1 (PDF 296 kb) [file 13238_2018_556_MOESM1_ESM.pdf]

Fig S1. Representative images showing immunostaining of differentiated EPS cells by EB assay. EB was immunostained by anti-FOXA2, anti-β-III TUBULIN, anti-GATA3, anti-EOMES and anti-CDX2 antibodies.

Fig S2. The generation of *IL6* knock-in EPS cells. Diagrams of the generation human *IL6* gene knock-in EPS cells. Primers for knock-in detection are indicated as pairs of arrows.

Fig S3. **(A)** Off-target effects of human *IL3* knock-in EPS cells. Left, T7E1 assay. Right, sequencing (20 clones per sample). **(B)** Off-target effects of human *IL6* knock-in EPS cells. Left, T7E1 assay. Right, sequencing (20 clones per sample).

Fig S4. Representative images showing immunostaining of EPS and EPS-*IL3* with lineage-specific markers. Error bar, 25 μm.

Fig S5. ES-IL3 cells cultured in 2i condition were generated using the same gene targeting strategy with EPS-IL3 cells. Two ES-IL3 sub-lines were selected for tetraploid complementation experiments at passage 20. 46 blastocysts were injected with ES-IL3 cells and surrogate mothers were sacrificed on day E10.5. (A) Placenta obtained from tetraploid assay using ES-IL3 cells, E10.5. (B) The development of EPS-IL3 and ES-IL3 cells in tetraploid blastocysts.

Fig. S1

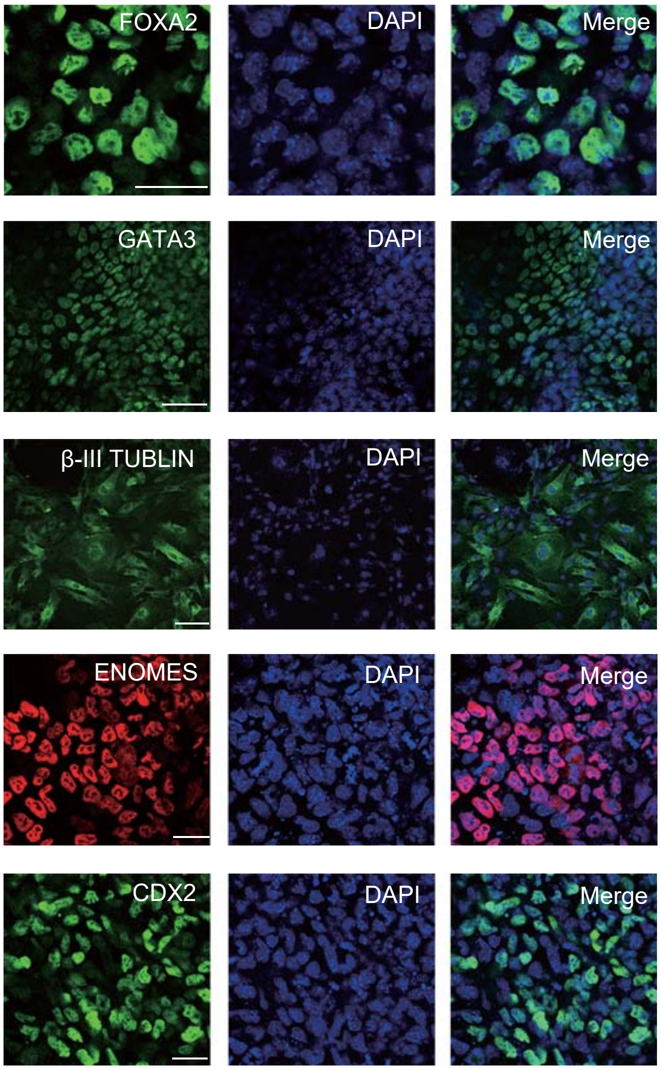

FOXA2
DAPI
Merge
GATA3
DAPI
Merge
β-III TUBLIN
DAPI
Merge
ENOMES
DAPI
Merge
CDX2
DAPI
Merge

Fig. S2

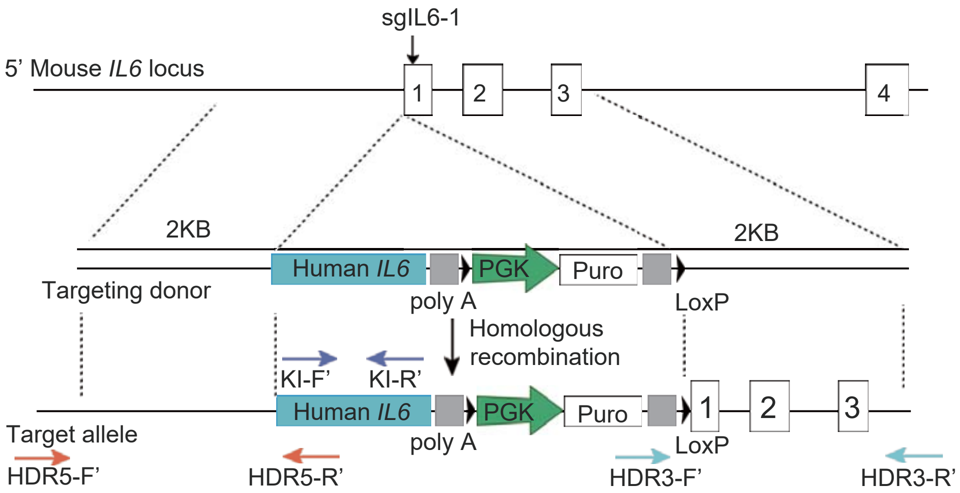

sgIL6-1
5' Mouse IL6 locus
1
2
3
4
2KB
2KB
Targeting donor
Human IL6
PGK
Puro
poly A
LoxP
Homologous recombination
KI-F'
KI-R'
Target allele
Human IL6
PGK
Puro
1
2
3
poly A
LoxP
HDR5-F'
HDR5-R'
HDR3-F'
HDR3-R'

Fig. S3

A Mouse *IL3* locus

5' GATGCTGGTGGTAGAGCTGGCAAGAACCATTGTCTCGTTCTGGTCCTCCAAGGGGTTCTGAAGAAG 3'

target PAM

offtarget 1 GTGGATACATTGTCTCGTTCAGG

offtarget 2 TTTTGGATATTGTCTCGTTCAGG

IL3 off-target 1

WT 3-1 3-7

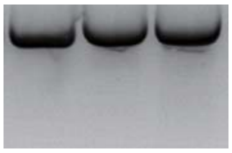

off-target 1

WT CTGGGGGAGGTGGATACATTGTCTCGTTCAGGGCTAGACACTCACA

3-1 OFF 1-1 CTGGGGGAGGTGGATACATTGTCTCGTTCAGGGCTAGACACTCACA

3-1 OFF 1-2 CTGGGGGAGGTGGATACATTGTCTCGTTCAGGGCTAGACACTCACA

3-7 OFF 1-1 CTGGGGGAGGTGGATACATTGTCTCGTTCAGGGCTAGACACTCACA

3-7 OFF 1-2 CTGGGGGAGGTGGATACATTGTCTCGTTCAGGGCTAGACACTCACA

IL3 off-target 2

WT 3-1 3-7

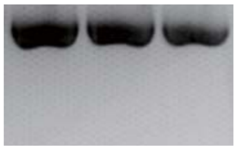

off-target 2

WT GTAATAAAATGCCAGTTTTGGATATTGTCTCGTTCAGGTGCAGTCCCA

3-1 OFF 2-1 GTAATAAAATGCCAGTTTTGGATATTGTCTCGTTCAGGTGCAGTCCCA

3-1 OFF 2-2 GTAATAAAATGCCAGTTTTGGATATTGTCTCGTTCAGGTGCAGTCCCA

3-7 OFF 2-1 GTAATAAAATGCCAGTTTTGGATATTGTCTCGTTCAGGTGCAGTCCCA

3-7 OFF 2-2 GTAATAAAATGCCAGTTTTGGATATTGTCTCGTTCAGGTGCAGTCCCA

B Mouse *IL6* locus

5' TGCCTCACACTCCTCTCTCACAGTCTCAATAGCTCCGCCAGAGGGCAAGGAACTGCCTTCACTTACT 3'

target PAM

offtarget 1 GTGATCCTAGCTCCGCCAGTGGG

offtarget 2 GTAAGCAGGGCTCCGCCAGAGGG

IL6 off-target 1

WT 6-3 6-9

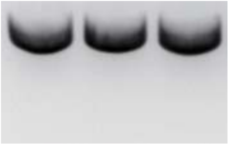

off-target 1

WT CAACTCTTTGTGATCCTAGCTCCGCCAGTGGGTGTGATGCAGTATC

6-3 OFF 1-1 CAACTCTTTGTGATCCTAGCTCCGCCAGTGGGTGTGATGCAGTATC

6-3 OFF 1-2 CAACTCTTTGTGATCCTAGCTCCGCCAGTGGGTGTGATGCAGTATC

6-9 OFF 1-1 CAACTCTTTGTGATCCTAGCTCCGCCAGTGGGTGTGATGCAGTATC

6-9 OFF 1-2 CAACTCTTTGTGATCCTAGCTCCGCCAGTGGGTGTGATGCAGTATC

IL6 off-target 2

WT 6-3 6-9

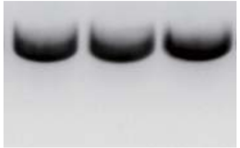

off-target 2

WT GAGCCAGTAAGCAGGGCTCCGCCAGAGGGGAGTGCTCAAAGGACA

6-3 OFF 2-1 GAGCCAGTAAGCAGGGCTCCGCCAGAGGGGAGTGCTCAAAGGACA

6-3 OFF 2-2 GAGCCAGTAAGCAGGGCTCCGCCAGAGGGGAGTGCTCAAAGGACA

6-9 OFF 2-1 GAGCCAGTAAGCAGGGCTCCGCCAGAGGGGAGTGCTCAAAGGACA

6-9 OFF 2-2 GAGCCAGTAAGCAGGGCTCCGCCAGAGGGGAGTGCTCAAAGGACA

Fig. S4

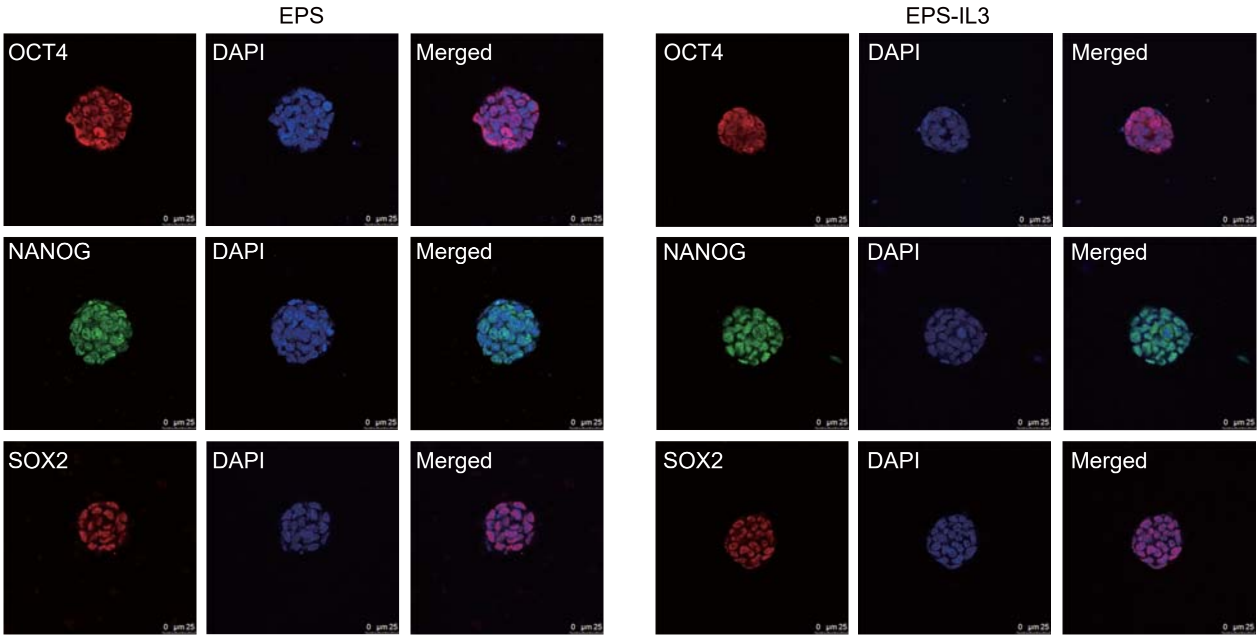

Fig. S5

A

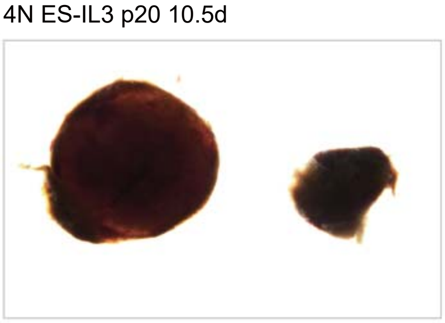

B

The development of ES-IL3 and EPS-IL3 cells in 4N tetraploid blastocysts.

	No.4N Blastocyst	Born/Blastocyst(%)	Alive/Born pups(%)
ES-IL3	46	0/46(0)	-
EPS-IL3	50	7/50(14)	5/7(71.4)
